# Annotation-free artificial intelligence for abdominal computed tomography anomaly detection

**DOI:** 10.1016/j.ebiom.2024.105497

**Published:** 2024-12-10

**Authors:** Jia Fu, Mengjie Fang, Zhuozhao Zheng, Di Dong

**Affiliations:** a Department of Radiology, Beijing Tsinghua Changgung Hospital, School of Clinical Medicine, Tsinghua University, Beijing, 102218, China; b Beijing Advanced Innovation Center for Big Data-Based Precision Medicine, School of Engineering Medicine, Beihang University, Beijing, 100191, China; c CAS Key Laboratory of Molecular Imaging, Institute of Automation, Chinese Academy of Sciences, Beijing, 100190, China

Abdominal computed tomography (CT) scans, which rapidly provide three-dimensional (3D) anatomical images, are crucial for diagnosing abdominal diseases, including early lesion detection, disease extent evaluation, and clinical treatment guidance. The growing volume of abdominal CT examinations has significantly increased the workload of radiologists,^1^ a trend expected to intensify in the future. This creates immense challenges for diagnostic efficiency and accuracy, potentially leading to a longer patient waiting time and increased risks of missed or incorrect diagnoses.

In recent years, advances in artificial intelligence (AI) technologies have demonstrated significant potential in medical image analysis.^2^^,^^3^ Training high-performing AI models generally relies on high-quality annotated data,^4^ either for building models from scratch or fine-tuning pre-trained ones. The process of medical data annotation is time-consuming, expensive, and dependent on highly specialized medical expertise, adding considerable pressure to radiologists who are already burdened with heavy clinical workloads.^5^ Thus, the ability to efficiently utilize existing data to develop robust and effective AI models under constrained resources has become a critical challenge and a priority in medical imaging AI research.

To tackle this challenge, Junya Sato and colleagues proposed to use a multi-organ segmentation model and an information extraction schema, creating a novel pathway for developing an abdominal CT anomaly detection model without manual annotations.^6^ The information extraction schema automatically extracts organ-specific disease information from free-text radiology reports to generate training labels, while the multi-organ segmentation model precisely segments regions of interest for individual organs in abdominal CT images. This approach significantly expands the volume of available training data without requiring manual annotations. The resulting model efficiently detects abnormalities in the liver, gallbladder, pancreas, spleen, and kidney, achieving the area under the ROC curve ranging from 0.838 to 0.903 in the external test cohort.

Currently, research directly utilizing radiology reports as labels for CT images remains limited. Achieving automated multi-organ segmentation and anomaly detection in the abdomen is particularly challenging due to the complexity of abdominal organs, which vary significantly in size, shape, and location. For instance, small organs like the pancreas and gallbladder exhibit substantial anatomical variability, with boundaries often influenced by adjacent structures. Additionally, imaging conditions, fat infiltration, or disease progression can blur organ boundaries. Furthermore, abdominal lesions are diverse and unevenly distributed, with some presenting only as subtle density changes, making detection and classification even more difficult.

To overcome these challenges, the research mentioned above employs a 3D full-resolution nnUNet model for high-precision multi-organ segmentation. A two-stage information extraction schema is designed to automatically identify abnormal features, diagnostic information, and modifying attributes through entity extraction and relationship construction, addressing challenges of processing unstructured information in radiology reports. Additionally, a multi-instance learning model combined with long short-term memory (LSTM) enhances the capture of complex lesion features, significantly improving anomaly detection sensitivity. By integrating these technical innovations and leveraging large-scale training datasets, efficient and accurate multi-organ anomaly detection in abdominal CT scans is successfully achieved. The experimental results confirm the reliability and accuracy of the method, paving the way for its application to other organs and diseases, with significant research and clinical potential.

Interestingly, it reveals that with limited data, high-quality manually annotated data yields better training outcomes due to its precision and reliability, providing clear learning targets for the model. However, as data volume grows, even auto-labeled data with minor errors can enhance model performance and eventually surpass manually annotated models. This phenomenon underscores the advantage of deep learning's reliance on data diversity and comprehensiveness, where large-scale datasets mitigate annotation errors and yield substantial performance gains. Consequently, automated labeling technologies not only show great potential in reducing annotation costs and time but also offer new insights into balancing data quality and quantity. For instance, in cross-institutional data-sharing scenarios, even if the data quality varies, sufficient data volume enables models to achieve robust generalization through joint training. Furthermore, automated labeling seamlessly integrates federated learning frameworks, where institutions can collaboratively train AI models without sharing sensitive raw data. This synergy enhances the diversity and scale of training datasets while preserving patient privacy. Federated learning, in turn, enables iterative improvements in automated labeling models, as globally trained models can refine local labeling processes.

In future, the integration of pre-trained large language models and multimodal large models into this framework promises finer-grained disease detection and detailed descriptions of anomalies.^7^ Furthermore, techniques like Chain-of-Thought (CoT), which allows step-by-step reasoning, and Retrieval-Augmented Generation (RAG), which dynamically retrieves relevant knowledge or reference data, can be incorporated to enhance both the diagnostic accuracy and clinical interpretability.^8^^,^^9^ CoT improves and demonstrates the logical reasoning process in complex decision-making tasks, while RAG ensures that models maintain contextual relevance during inference by referencing external data sources, providing effective support for decision-making. The introduction of these techniques can further expand the model's utility in real-world scenarios while providing innovative solutions for multimodal data integration and complex medical tasks.

## Contributors

All authors contributed to conceptualization, writing, reviewing, editing and have read and approved the published version of the manuscript.

## Declaration of interests

The authors declare no conflict of interest.
